# Effectiveness of a Multicomponent Digital Therapeutic Program on Inhaled Medication Adherence in Chronic Obstructive Pulmonary Disease: Pilot Randomized Controlled Trial

**DOI:** 10.2196/98870

**Published:** 2026-11-06

**Authors:** Hanna Wu, Fen Dong, Yanan Cui, Danjing Hu, Yushi Zhang, Tianqi Peng, Yaodie Peng, Xiang Li, Yong Li, Xiaopan Li, Yongxia Chen, Qingjie Yu, Hongtao Niu, Ting Yang

**Affiliations:** 1 Peking University China-Japan Friendship School of Clinical Medicine, Yinghua Street, Beijing, 100029, China, 86 8420-5566; 2 National Center for Respiratory Medicine; State Key Laboratory of Respiratory Health and Multimorbidity; National Office for Chronic Respiratory Disease Prevention and Control; National Clinical Research Center for Respiratory Diseases; Institute of Respiratory Medicine, Chinese Academy of Medical Sciences & Peking Union Medical College; Department of Pulmonary and Critical Care Medicine, Center of Respiratory Medicine, China-Japan Friendship Hospital, Beijing, China; 3 Chinese Academy of Medical Sciences & Peking Union Medical College, Beijing, China; 4 Tianjin Center for Medical Devices Evaluation and Inspection, Tianjin, China

**Keywords:** digital therapeutics, chronic obstructive pulmonary disease, COPD, adherence, electronic inhaler monitoring

## Abstract

**Background:**

Poor medication adherence is a critical impediment to effective chronic obstructive pulmonary disease (COPD) management and remains challenging to track in routine care. Digital therapeutic interventions may improve chronic disease management through remote monitoring and patient education. However, most existing digital interventions focus on individual components of disease management, and evidence from longer-term pragmatic randomized controlled trials evaluating integrated digital therapeutics in routine clinical practice remains limited.

**Objective:**

This study aimed to evaluate the effectiveness of a multicomponent digital therapeutic program combining electronic inhaler monitoring (EIM) with behavioral support in improving medication adherence and symptom-related outcomes among patients with COPD prescribed pressurized metered-dose inhalers, and to assess patient engagement and implementation in routine clinical practice.

**Methods:**

In this prospective, open-label, unblinded, randomized controlled trial, patients with COPD prescribed pressurized metered-dose inhalers were enrolled and randomized in a 2:1 ratio to either the intervention group or the control group. All participants received an EIM device to objectively record inhaler use throughout the 24-week study period. Patients in the intervention group also received personalized medication reminders from the EIM, adherence feedback, educational content, and clinician-supported follow-up through an integrated digital management platform based on EIM-derived data, whereas the control group received usual care. The primary outcome was medication adherence over 24 weeks. Secondary outcomes included exacerbations, COPD symptoms, quality of life, and lung function.

**Results:**

A total of 158 participants were randomized, including 106 in the intervention group and 52 in the control group. The median monitoring coverage was 161.5 (IQR 86.3‐168.0) days during the follow-up period. The self-reported adherence measured using the Test of Adherence to Inhalers showed no significant between-group differences at any follow-up time point. However, the electronically monitored adherence was significantly higher in the intervention group than in the control group (59.5% vs 33.1%, *P*=.009). Adherence declined over time in both groups, decreasing from 85.3% during weeks 0 to 4 to 43.0% during weeks 12 to 24 in the intervention group and from 76.8% to 11.9% in the control group over the same intervals, resulting in a progressively larger between-group difference over follow-up. No significant differences were observed in exacerbations, health-related quality of life, symptom burden, lung function, or mental health outcomes. In an exploratory analysis, a significantly greater proportion of participants in the intervention group achieved the minimal clinically important difference in the COPD Assessment Test score than in the control group (odds ratio 2.21, 95% CI 1.11‐4.41; *P*=.02).

**Conclusions:**

The multicomponent digital therapeutic program significantly improved objective medication adherence in patients with COPD over 24 weeks. While most clinical outcomes did not differ significantly between groups, the higher proportion of participants achieving a clinically meaningful improvement in the COPD Assessment Test score warrants further investigation.

## Introduction

Chronic obstructive pulmonary disease (COPD) is a leading cause of morbidity and mortality worldwide and imposes a substantial burden on health care systems [1-3]. Inhaled pharmacotherapy remains the cornerstone of COPD management. However, suboptimal adherence to inhaled therapy remains highly prevalent and is a major barrier to effective disease control, contributing to an increased risk of exacerbations and a higher symptom burden [4,5]. Adherence is influenced by multiple interrelated factors at the patient, inhaler, and health care provider levels, including age, cognitive and physical abilities, health literacy, behavioral determinants, inhaler complexity and usability, and health care professionals’ competency and capacity to provide appropriate inhaler education. The multidimensional nature of these factors highlights the complexity of addressing medication nonadherence in routine COPD care [6,7]. Monitoring adherence to inhaled therapy is also challenging in routine clinical practice. Traditional approaches such as patient self-report and pharmacy refill records are prone to substantial inaccuracies and may overestimate adherence due to recall bias or demonstration of idealized inhaler technique, failing to capture real-world medication use [8].

Recent advances in digital health have introduced digital therapeutic interventions designed to improve chronic disease management through behavioral support, remote monitoring, and patient education. In respiratory diseases, digital therapeutic programs typically include digital inhaler platforms and mobile health apps [9,10]. Digital inhaler monitoring devices attached to inhalers, which can objectively record medication use, provide automated reminders, and enable personalized adherence feedback, have been shown to improve adherence, enhance disease control, and reduce health care burden in chronic airway diseases in previous trials. However, most existing studies have focused on asthma and have evaluated individual components, such as adherence reminders, inhaler monitoring, or telehealth consultations. In COPD, few interventions have combined objective inhaler medication monitoring with patient self-management tools and remote clinician monitoring within a single platform [11-17]. Evidence from randomized trials evaluating integrated digital therapeutic programs in COPD remains limited, particularly in real-world clinical settings.

To address this gap, we developed a multicomponent digital therapeutic program integrating an electronic inhaler monitoring (EIM) device with a mobile management app and conducted the DIGI-COPD (Effectiveness of a Multi-Component Digital Therapeutic Program on Inhaled Medication Adherence in COPD) trial to evaluate whether this intervention could improve inhaled medication adherence and clinical outcomes in patients with COPD.

## Methods

### Study Design

This prospective, open-label, unblinded pilot randomized trial was conducted at the respiratory outpatient clinic of China-Japan Friendship Hospital, Beijing, China. Patients were recruited through physician recommendation during outpatient visits or self-referral in response to advertisements posted in the outpatient area. All potential participants underwent eligibility screening by the research team, which included review of medical records and spirometry results obtained within the previous 6 months, and were invited to an introductory meeting to review the study details and ask questions. Those who met the eligibility criteria and agreed to participate provided written informed consent, and baseline information was then collected.

At baseline, demographic and clinical characteristics, medical and smoking history, and exacerbation history of the participants were collected. All participants completed the modified Medical Research Council (mMRC), COPD Assessment Test (CAT), St George’s Respiratory Questionnaire for Chronic Obstructive Pulmonary Disease (SGRQ-C), Patient Health Questionnaire-9 (PHQ-9), Generalized Anxiety Disorder-7 (GAD-7), and Test of Adherence to Inhalers (TAI) questionnaires [18]. Trained assessors evaluated participants’ inhaler technique using the metered-dose inhaler score [19] and performed physical examination (height, weight, waist and hip circumference, blood pressure, resting pulse rate, and oxygen saturation). Follow-up assessments were conducted at weeks 4, 12, and 24. This study was reported in accordance with the CONSORT (Consolidated Standards of Reporting Trials) guidelines (Checklist 1).

### Participants and Randomization

Patients were eligible if they (1) fully understood and were willing to sign the written informed consent, (2) were local residents diagnosed with COPD by a physician, (3) were aged 40 years or older, (4) were prescribed a metered-dose inhaler as long-term treatment, and (5) were able to use a smartphone app after training. COPD diagnosis was confirmed during screening by reviewing medical records and spirometry results obtained within the previous 6 months, including a documented postbronchodilator FEV_1_/FVC (forced expiratory volume in 1 second/forced vital capacity) <0.70.

Participants were randomized in a 2:1 ratio (intervention:control) stratified into 2 strata according to baseline CAT score (<10 vs ≥10) using a computer-generated randomization sequence. Neither researchers nor participants were blinded to group assignment.

### Sample Size

The initial sample size was estimated based on a previous questionnaire conducted by our research group, in which 258 of 737 (35.01%) patients with COPD were classified as having good adherence. Assuming a 15% absolute increase in the proportion of patients with good adherence in the intervention group at week 24, with an α of 0.05 and 90% power, the estimated sample size was 400 participants in the intervention group and 200 in the control group, allowing for approximately 20% loss to follow-up. However, as this was a pilot study, recruitment was conducted within a predefined study period and was constrained by available funding and research personnel.

### Intervention

The multicomponent digital therapeutic program comprised an EIM and a remote management platform for both patients and clinicians, supporting both patient self-management and clinician oversight (Figure 1A).

The same EIM was compatible with any pressurized metered-dose inhaler (pMDI) device regardless of whether the prescribed regimen was dual bronchodilation therapy, consisting of a long-acting beta-agonist and a long-acting muscarinic antagonist, or triple therapy, consisting of a long-acting beta-agonist, a long-acting muscarinic antagonist, and inhaled corticosteroids, as it was externally attached to the pMDI. It passively recorded each inhalation event with a time stamp and transmitted the data via a cellular network to the patient’s mobile phone. The adherence data were then synchronized with the patient app and, with patient consent, transmitted to a cloud-based data center. A built-in buzzer delivered audible reminders at scheduled dosing times for the intervention group. The device emitted a daily “heartbeat” signal to confirm proper functioning (Figure 1B). The safety of the device has been tested by the China Mobile Electronic Component Testing Center.

The management app enabled patients to visualize adherence data through graphs and tables, generate monthly adherence reports, complete a daily COPD Exacerbation Recognition Tool (CERT) diary [20], and record vital signs. Additional features included accessing step counts and weather information, setting medication reminders, self-reporting of CAT and mMRC scores, and viewing educational content. Clinicians could remotely monitor patient data via mobile or desktop devices, identify high-risk individuals, and initiate timely, individualized communication (Figure 1C).

Participants in the intervention group received an EIM with a reminder paired with a mobile management app, which delivered personalized reminders and adherence feedback based on EIM data. They were instructed to complete a daily CERT diary and record vital signs in the app, which displayed trends and generated monthly reports on adherence, physiological parameters, and symptoms. Participants in the control group received an EIM without reminders, solely for recording inhaler use. They also had the app installed solely to relay EIM data, without access to adherence feedback or educational content, or clinician access for review. Both groups continued to receive usual care from their physicians at each scheduled follow-up visit.

**Figure 1.**
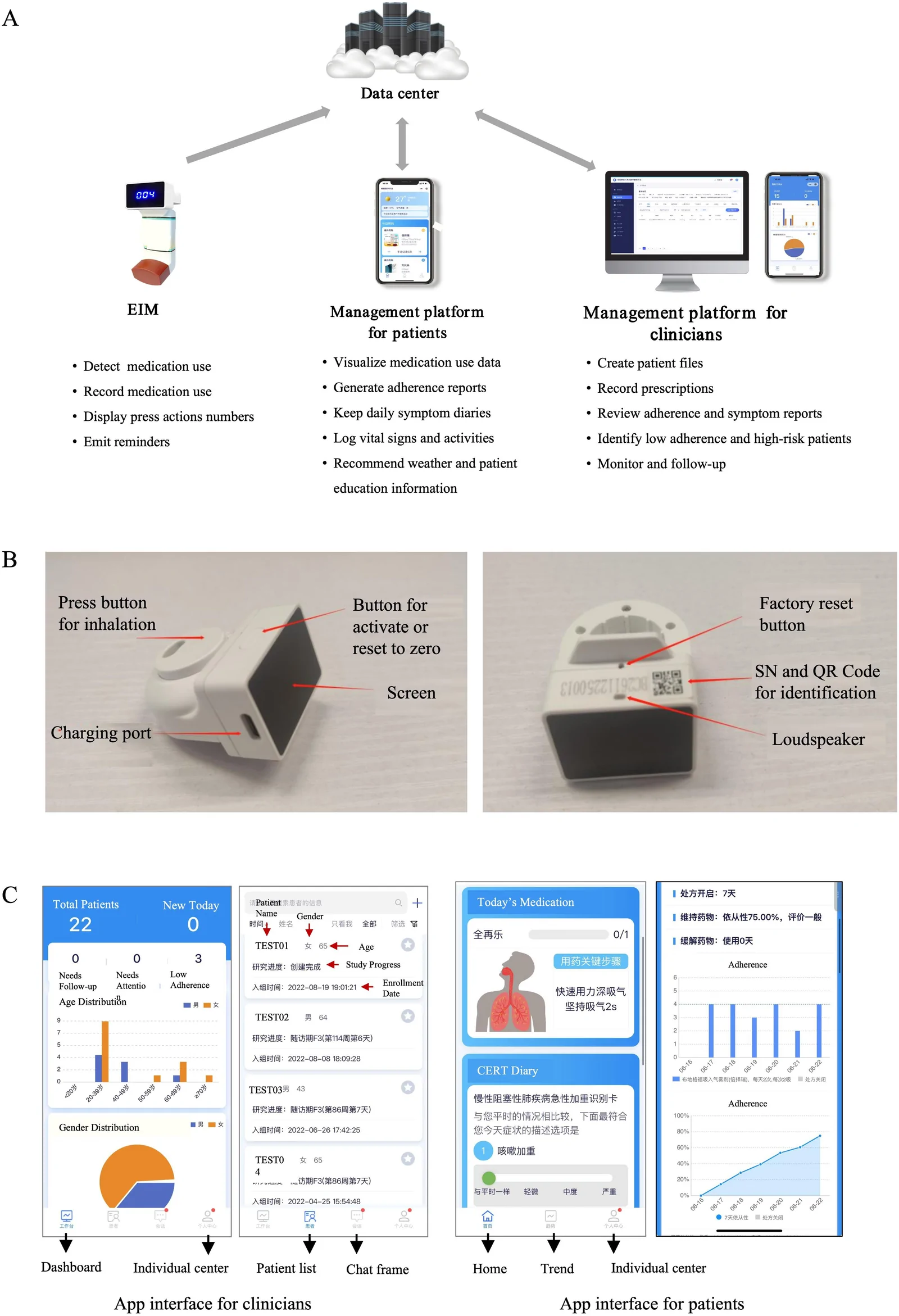
Multicomponent digital therapeutic program. (A) Framework of the program. (B) Appearance of the electronic inhaler monitoring (EIM) device. (C) User interface of the management app. SN: serial number.

### Outcome Measures

The primary outcomes were the proportion of participants with good adherence (TAI score ≥46) and the average daily adherence (calculated as the ratio of actual inhalations over prescribed inhalations) recorded by EIM during the study period. The TAI questionnaire assessed self-reported adherence during the preceding week and was administered at baseline and each scheduled follow-up visit. Adherence was continuously recorded by the EIM and summarized as the average daily adherence over the corresponding follow-up intervals (weeks 0‐4, 4‐12, and 12‐24). The secondary outcomes included CAT score, mMRC score, PHQ-9 score, GAD-7 score, lung function, and the number of exacerbations between groups.

### Statistical Analysis

All 158 randomized participants were included in the intention-to-treat analysis for primary and secondary outcomes. Continuous data were presented as mean with SD or median with IQR and analyzed using 2-tailed Student *t* tests or Mann-Whitney *U* tests as appropriate, and categorical data were presented as numbers with percentages and analyzed using the chi-square test or Fisher exact test. Two-sided tests were used throughout, and a value of *P*<.05 was considered statistically significant. Statistical analyses were conducted using IBM SPSS (version 29.0.1.0) for data management and descriptive statistics, R (version 4.4.2; R Foundation for Statistical Computing) for linear mixed-effects modeling and advanced analyses, and GraphPad Prism (version 10.4.1) for data visualization.

The primary outcome was analyzed using a linear mixed-effects model, with follow-up adherence measurements as the dependent variable, and group, time, and their interaction as fixed effects. A random intercept was included to account for within-participant correlation, and an unstructured covariance matrix was used for repeated measures over unequally spaced visits. Secondary outcomes were analyzed as follows: acute exacerbations using negative binomial regression; lung function and SGRQ scores using linear mixed models (after the Levene test indicated heterogeneity of variance, *P*=.046); and PHQ-9, GAD-7, mMRC, and CAT scores using linear mixed models. All models were adjusted for corresponding baseline values.

Subgroup analyses for mMRC and CAT scores examined potential effect modification by including group, time, subgroup, and their 3-way interaction terms in linear mixed models, with a significance threshold of *P*<.10 for interaction terms and not adjusted for multiple comparisons because it was considered exploratory. For other secondary outcomes, potential effect modification was examined using linear regression models that included group, subgroup, and their interaction term.

### Ethical Considerations

The study was approved by the China-Japan Friendship Hospital Ethics Committee (approval number 2022-KY-129) and was conducted in accordance with the Declaration of Helsinki (1975, as revised in 2008) and applicable Chinese regulations. Written informed consent was obtained from all participants before enrollment. Participant data were deidentified prior to analysis and stored on secure password-protected servers accessible only to authorized study personnel. Participants did not receive any financial compensation for participation. As appreciation for their time and contribution, participants were offered 1 complimentary lung function test at the 24-week follow-up and permanent access to the EIM device and integrated digital management platform after study completion, regardless of study group.

## Results

### Participant Characteristics

Participant recruitment was conducted between March 6, 2023, and January 31, 2024, and follow-up was completed on July 17, 2024. In the context of the COVID-19 pandemic during the recruitment period, a total of 158 eligible participants provided written informed consent by the end of the predefined study period, with 106 assigned to the intervention group and 52 to the control group. Participants continued their physician-prescribed inhaled therapy throughout the study. A total of 19 participants in the intervention group and 10 in the control group withdrew during the study, of whom 11 discontinued due to pMDI cessation (Figure 2). Among the remaining participants, 2 in the intervention group (1 switched from dual bronchodilation to triple therapy and 1 switched from triple therapy to dual bronchodilation) and 1 in the control group (switched from dual bronchodilation to triple therapy) had their inhaled regimens switched by their physicians during the study, without changing the pMDI device. No other changes in inhaled therapy were documented.

Baseline characteristics were similar between groups. A total of 129 (81.6%) participants were male, and the mean age was 64 (SD 8.30) years. The mean number of acute exacerbations in the previous year (including mild, moderate, and severe) was 0.95 (SD 1.34), and 72.2% (n=114) of participants were classified as having good adherence based on the self-reported TAI questionnaire (Table 1).

**Figure 2.**
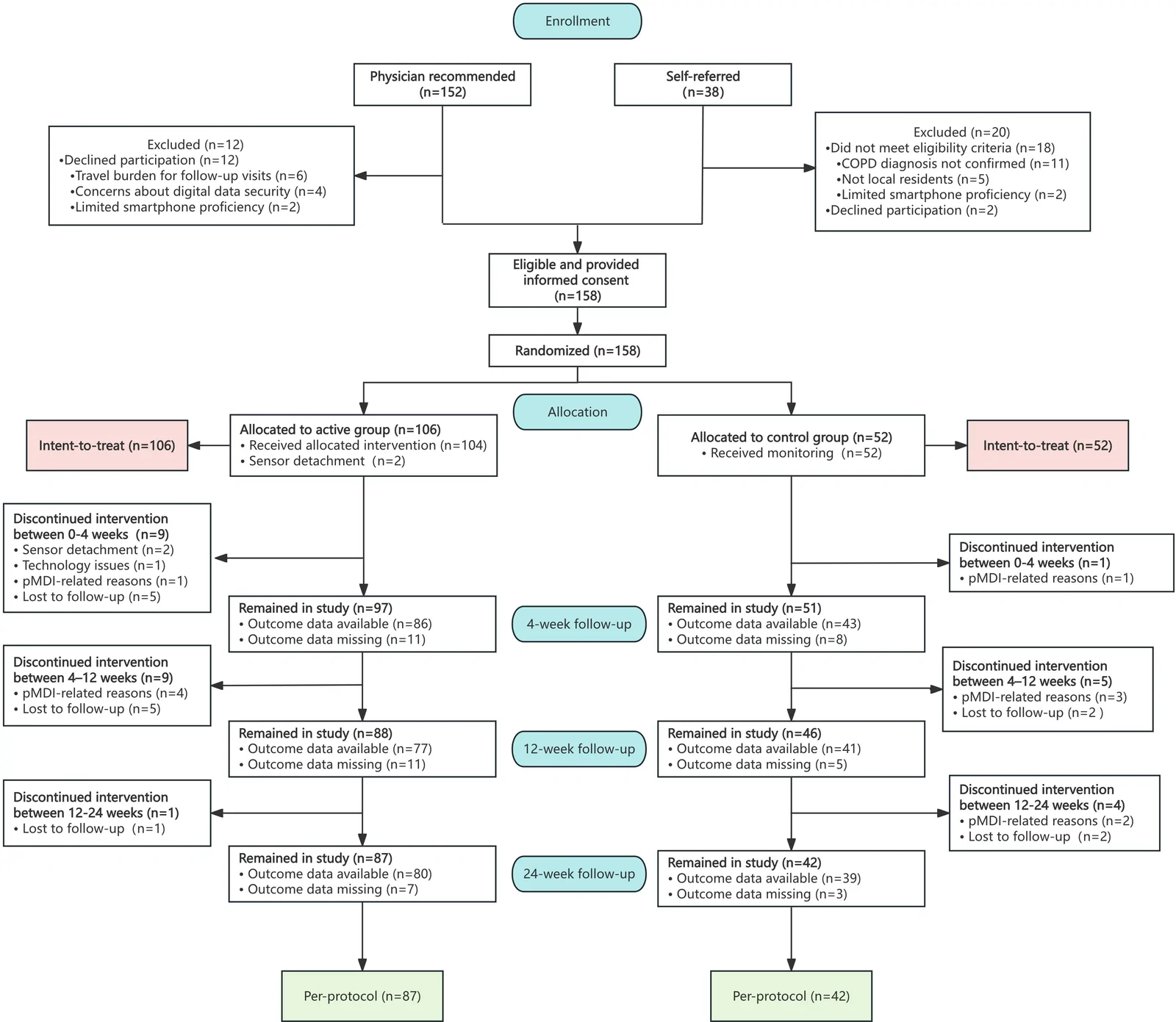
Flow diagram of the study. COPD: chronic obstructive pulmonary disease; pMDI: pressurized metered-dose inhaler.

**Table 1.** Baseline characteristics of intervention and control participants (N=158).

Variables	All participants (N=158)	Intervention group (n=106)	Control group (n=52)	*P* value
Age (y), mean (SD)	64.08 (8.30)	63.30 (8.74)	65.65 (7.16)	.09
Sex (male), n (%)	129 (81.6)	85 (80.2)	44 (84.6)	.66
BMI (kg/m^2^), mean (SD)	24.33 (4.03)	24.49 (4.03)	23.98 (4.02)	.45
Education level, n (%)				.90
No formal education	6 (3.7)	5 (4.7)	1 (1.9)	
Primary school	16 (10.1)	10 (9.4)	6 (11.5)	
Junior high school	52 (32.9)	35 (33.0)	17 (32.6)	
Senior high school	43 (27.2)	27 (25.4)	16 (30.7)	
College/associates degree	29 (18.3)	20 (18.8)	9 (17.3)	
Postgraduate degree	12 (7.5)	9 (8.4)	3 (5.7)	
Health insurance type, n (%)				.73
Urban employee basic	86 (54.4)	56 (52.8)	30 (57.6)	
New rural cooperative	35 (22.1)	22 (20.7)	13 (25.0)	
Urban resident basic	24 (15.1)	19 (17.9)	5 (9.6)	
Urban and rural resident	3 (1.8)	2 (1.8)	1 (1.9)	
Private	10 (6.3)	7 (6.6)	3 (5.7)	
Smoking status, n (%)				.40
Never	42 (26.6)	31 (29.2)	11 (21.2)	
Former	65 (41.1)	40 (37.7)	25 (48.1)	
Current	51 (32.3)	35 (33.0)	16 (30.8)	
Smoking pack-year history for smokers, mean (SD)	19.49 (5.30)^a^	19.49 (5.67)^b^	19.49 (4.64)^c^	.10
FVC^d^ (L), mean (SD)	3.07 (0.90)	3.09 (0.90)	3.03 (0.88)	.71
FEV_1_^e^ (L), mean (SD)	1.68 (0.70)	1.71 (0.71)	1.63 (0.68)	.51
FEV_1_ predictive (%), mean (SD)	60.66 (22.96)	60.59 (8.74)	60.78 (23.86)	.96
FEV_1_/FVC (%), mean (SD)	53.88 (13.08)	54.24 (12.39)	53.14 (14.45)	.63
PEF^f^ (L/s), mean (SD)	4.70 (2.17)	4.79 (2.11)	4.52 (2.28)	.48
GOLD^g^ stage, n (%)				.71
I	30 (18.9)	18 (16.9)	12 (23.0)	
II	66 (41.7)	47 (44.3)	19 (36.5)	
III	41 (25.9)	27 (25.4)	14 (26.9)	
IV	13 (8.2)	8 (7.5)	5 (9.6)	
Prior year exacerbation history				
Mean (SD)	0.95 (1.34)	1.00 (1.39)	0.85 (1.26)	.42
At least 1, n (%)	70 (44.3)	49 (46.2)	21 (40.4)	.49
Inhaled prescription, n (%)				.92
ICS^h^/LAMA^i^/LABA^j^	128 (81.0)	85 (80.2)	42 (80.8)	
LAMA/LABA	30 (19.0)	21 (19.8)	10 (19.2)	
mMRC^k^ score ≥2, n (%)	76 (48.1)	51 (48.1)	25 (48.1)	.10
CAT^l^ score ≥10, n (%)	59 (37.3)	40 (37.7)	19 (36.5)	.88
TAI^m^ score				
Mean (SD)	45.96 (6.84)	45.74 (7.09)	45.60 (6.36)	.90
≥46, n (%)	114 (72.2)	78 (73.6)	36 (69.2)	.57
SGRQ-C^n^ total score, mean (SD)	38.70 (19.18)	38.83 (18.34)	38.42 (20.98)	.90
PHQ-9^o^ score				
Mean (SD)	1.56 (2.95)	1.68 (3.25)	1.33 (2.23)	.48
≤4, n (%)	143 (90.5)	96 (90.6)	47 (90.4)	>.99
GAD-7^p^ score				
Mean (SD)	0.87 (2.16)	0.79 (2.09)	1.04 (2.31)	.50
≤4, n (%)	149 (94.3)	101 (95.3)	48 (92.3)	.69
Comorbidity, n (%)				
None	27 (17.0)	17 (16.0)	10 (19.2)	.62
Tuberculosis	11 (6.9)	8 (7.5)	3 (5.7)	.71
Cardiovascular disease	25 (15.8)	18 (16.9)	7 (13.4)	.62
Hypertension	61 (38.6)	42 (39.6)	19 (36.5)	.74
Diabetes	18 (11.3)	14 (13.2)	4 (7.6)	.29
Osteoporosis	14 (8.8)	9 (8.4)	5 (9.6)	.74
Anxiety or depressive disorder	3 (1.8)	2 (1.8)	1 (1.9)	>.99

a n=116.

b n=75.

c n=41.

d FVC: forced vital capacity.

e FEV_1_*:* forced expiratory volume in 1 second.

f PEF: peak expiratory flow rate.

g GOLD: Global Initiative for Chronic Obstructive Lung Disease.

h ICS: inhaled corticosteroid.

i LAMA: long-acting muscarinic antagonist.

j LABA: long-acting beta-agonist.

k mMRC: modified Medical Research Council.

l CAT: COPD Assessment Test.

m TAI: Test of Adherence to Inhalers.

n SGRQ-C: St George’s Respiratory Questionnaire for Chronic Obstructive Pulmonary Disease.

o PHQ-9: Patient Health Questionnaire–9.

p GAD-7: Generalized Anxiety Disorder–7.

### Adherence (Primary Outcome)

Except for 2 participants in the intervention group with sensor detachment, 156 participants had at least one valid daily adherence record. The median number of valid monitoring days was 161.5 (IQR 86.3‐168.0). TAI scores were collected at 3 follow-up visits, with response rates of 82.9% (n=131; 88 intervention vs 43 control), 75.3% (n=119; 77 vs 42), and 79.1% (n=125; 85 vs 40). No statistically significant differences were observed between groups in the proportion of participants classified as having good TAI adherence at any visits (Table 2). Linear mixed-effects modeling indicated that neither the main effect of group (*P*=.64), time within group (*P*=.051), nor the group × time interaction (*P*=.66) reached statistical significance.

**Table 2.** Self-reported and electronically measured adherence at weeks 0 to 4, 4 to 12, and 12 to 24 (N=158).

Adherence	0 to 4 weeks			4 to 12 weeks			12 to 24 weeks		
	Intervention group (n=106)	Control group (n=52)	*P* value	Intervention group (n=106)	Control group (n=52)	*P* value	Intervention group (n=106)	Control group (n=52)	*P* value
TAI^a^ score ≥46, n (%)	92 (86.8)	41 (78.8)	.20	88 (83.0)	42 (80.0)	.73	77 (72.6)	38 (73.1)	.95
Electronic adherence^b^ (%), median (IQR)	85.27 (55.36‐95.54)	76.79 (46.65‐85.49)	.04^c^	74.11 (31.70‐89.96)	31.47 (8.59‐76.00)	.004^c^	43.01 (2.53‐87.57)	11.90 (0.00‐71.73)	.03^c^

a TAI*:* Test of Adherence to Inhalers. TAI was assessed at weeks 4, 12, and 24, with each assessment reflecting self-reported adherence during the preceding 1 week.

b Electronic adherence was continuously recorded by the electronic inhaler monitoring and averaged over the corresponding intervals (weeks 0‐4, 4‐12, and 12‐24).

c Statistically significant values (*P*<.05).

Over the 24-week study period, the electronically measured medication adherence was significantly higher in the intervention group than the control group (59.52% vs 33.11%; *P*=.009; Figure 3A). The between-group difference increased over time: 85.27% vs 76.79% during weeks 0 to 4 (*P*=.04), 74.11% vs 31.47% during weeks 4 to 12 (*P*=.004), and 43.01% vs 11.90% during weeks 12 to 24 (*P*=.03; Table 2 and Figure 3B). The proportion of days with 100% of prescribed inhalations taken was also higher in the intervention group than in the control group (31.5% vs 16.7%; *P*=.007). Linear mixed-effects modeling revealed a significant main effect of group (*P*=.004) and time within groups (*P*<.001), whereas the group × time interaction did not reach statistical significance (*P*=.24).

**Figure 3.**
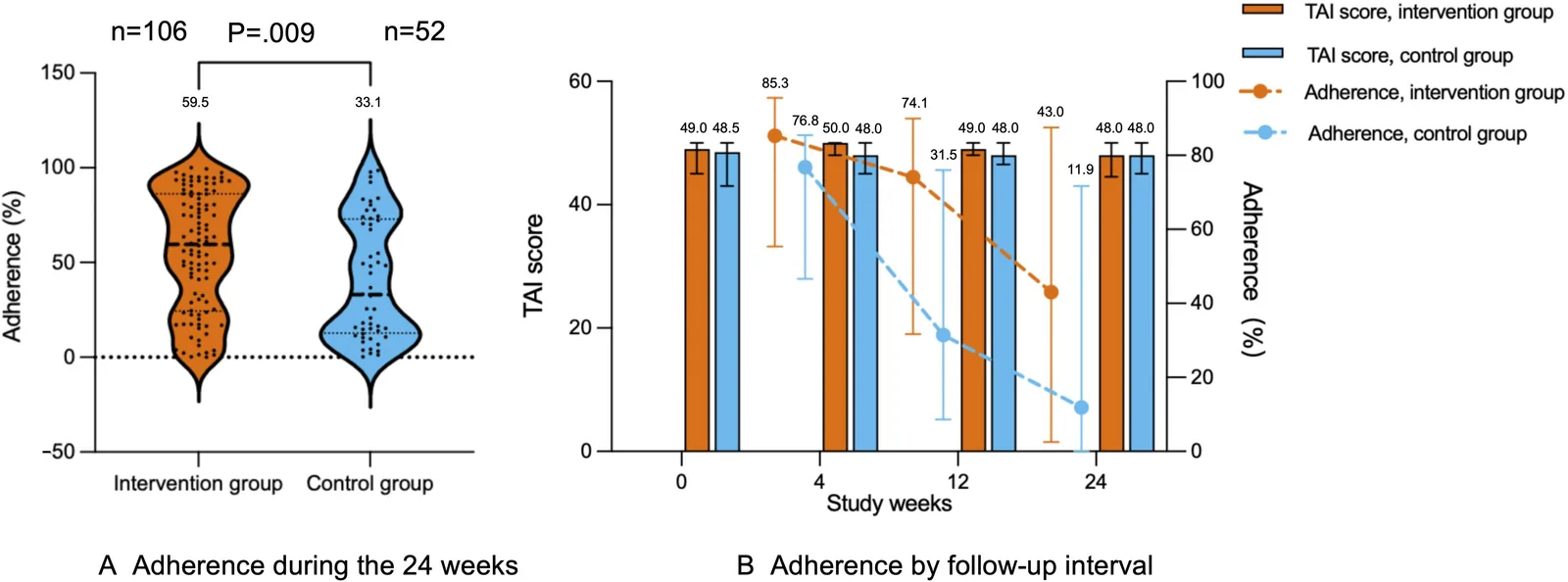
Adherence (median and IQR) over 24 weeks. (A) Violin plot of electronically measured adherence averaged over 24 weeks. (B) Test of Adherence to Inhalers (TAI) score at baseline, weeks 4, 12, and 24 and electronically measured adherence averaged over weeks 0 to 4, 4 to 12, and 12 to 24.

### COPD Assessments (Secondary Outcomes)

No statistically significant between-group differences were observed for SGRQ-C scores, PHQ-9 score, GAD-7 score, mMRC grade, CAT score, or lung function at week 24 (Table 3).

No significant between-group differences were detected for continuous CAT and mMRC scores either; however, longitudinal analyses showed significant changes over time in both groups (main effect of time, both *P*<.001). Compared with baseline, the mean change in CAT score was −7.09 (95% CI −10.55 to −3.62) at week 4,−8.60 (95% CI −12.03 to −5.17) at week 12, and −8.72 (95% CI −12.18 to −5.25) at week 24 (all *P*<.001). The mean change in mMRC grade was −0.59 (95% CI −0.97 to −0.22), −0.87 (95% CI −1.25 to −0.50), and −0.93 (95% CI −1.32 to −0.54) at weeks 4, 12, and 24 (Figure 4). Exploratory generalized estimating equation analysis showed that the intervention group had higher odds of achieving the minimal clinically important difference in CAT scores over follow-up (odds ratio 2.21, 95% CI 1.11‐4.41; *P*=.02), with a significant group × time interaction (*P*<.001). However, no individual follow-up time point remained statistically significant after Bonferroni correction for multiple comparisons (Figure 5).

**Table 3.** Secondary outcomes at week 24.

Variables	Intervention group, β^a^ (95% CI)	Control group, β (95% CI)	*P* value
SGRQ-C^b^ total score	20.29 (18.34 to 22.23)	23.38 (20.51 to 26.25)	.08
SGRQ-C symptoms score	34.89 (31.46 to 38.33)	40.10 (35.03 to 45.17)	.10
SGRQ-C activity score	29.02 (26.01 to 32.02)	31.49 (27.06 to 35.93)	.36
SGRQ-C impact score	10.07 (8.41 to 11.74)	12.84 (10.38 to 15.30)	.07
PHQ-9^c^ score	0.75 (0.29 to 1.20)	0.22 (−0.46 to 0.90)	.21
GAD-7^d^ score	0.50 (0.06 to 0.94)	0.09 (−0.56 to 0.75)	.31
mMRC^e^ score	0.987 (0.86 to 1.12)	0.926 (0.73 to 1.12)	.31
CAT^f^ score	6.36 (5.57 to 7.15)	7.11 (5.95 to 8.28)	.29
FVC^g^ (L)	3.25 (3.13 to 3.38)	3.16 (2.98 to 3.33)	.37
FEV_1_^h^ (L)	1.88 (1.78 to 1.98)	1.77 (1.63 to 1.91)	.22
FEV_1_ predictive (%)	65.25 (61.93 to 68.58)	62.26 (57.51 to 67.02)	.31
FEV_1_/FVC (%)	57.5 (55.0 to 60.0)	54.6 (51.0 to 58.2)	.19
PEF^i^ (L/s)	5.53 (5.21 to 5.85)	5.24 (4.78 to 5.69)	.30

a β: regression coefficient.

b SGRQ-C: St George’s Respiratory Questionnaire for Chronic Obstructive Pulmonary Disease.

c PHQ-9: Patient Health Questionnaire-9.

d GAD-7: Generalized Anxiety Disorder-7.

e mMRC: modified Medical Research Council.

f CAT: COPD Assessment Test.

g FVC: forced vital capacity.

h FEV_1_: forced expiratory volume in 1 second.

i PEF: peak expiratory flow rate.

**Figure 4.**
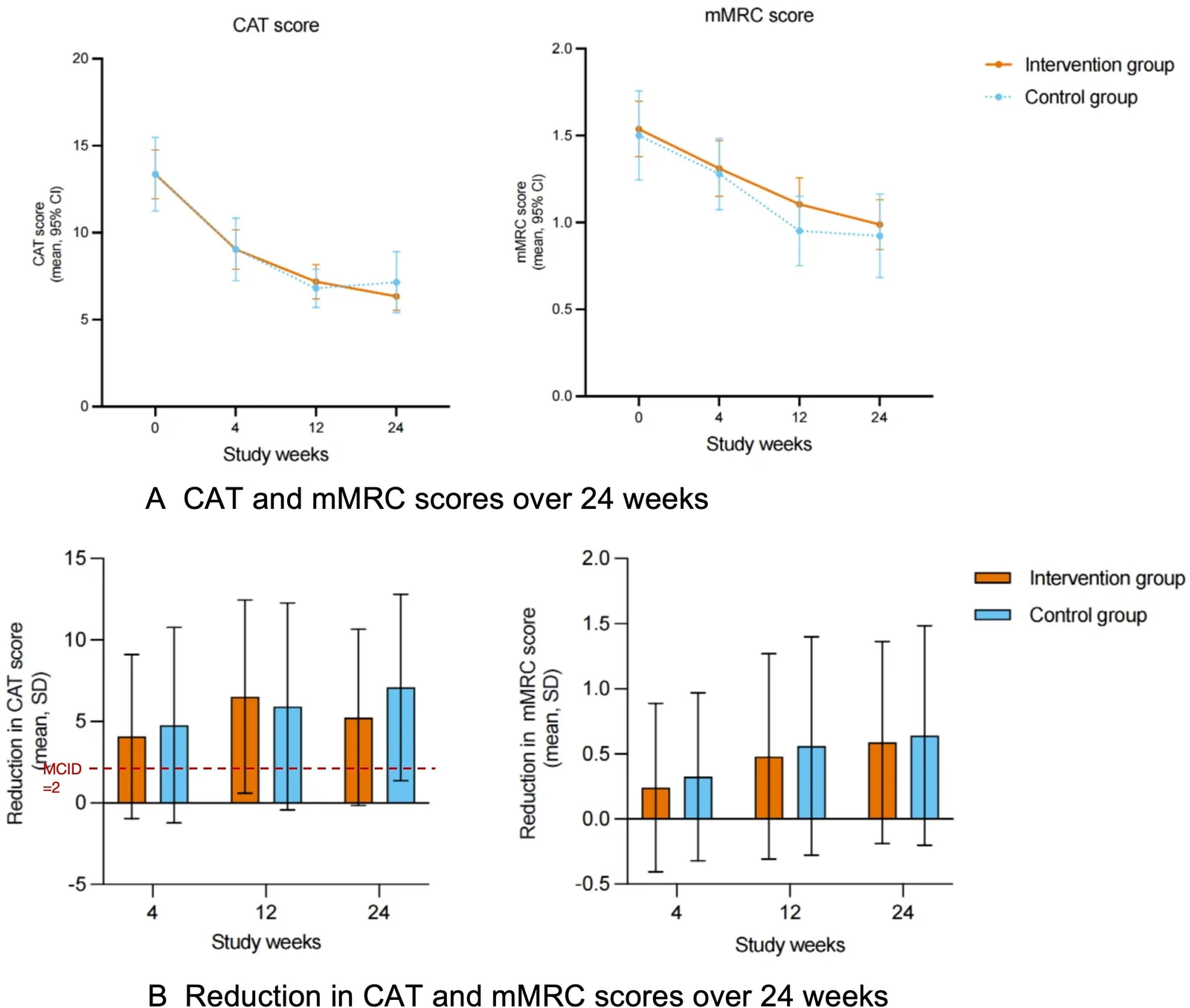
(A) COPD Assessment Test (CAT) and modified Medical Research Council (mMRC) scores over 24 weeks. Mean scores at weeks 4, 12, and 24. (B) Mean reduction from baseline at weeks 4, 12, and 24. COPD: chronic obstructive pulmonary disease; MCID: minimal clinically important difference.

During the 24-week follow-up, the mean number of moderate-to-severe exacerbations was 0.11 (SD 0.89) in the intervention group and 0.44 (SD 0.90) in the control group, with 8 (7.5%) and 10 (19.2%) participants, respectively, experiencing at least one event. After adjustment for baseline exacerbation history, negative binomial regression showed no statistically significant between-group differences in moderate-to-severe exacerbations (incidence rate ratio 0.68, 95% CI 0.24‐1.94; *P*=.47) or severe exacerbations (incidence rate ratio 0.54, 95% CI 0.15‐2.00; *P*=.36; Figure 5). Baseline exacerbation history was a strong predictor of subsequent events, with each additional exacerbation at baseline associated with a 3.69-fold higher risk of exacerbation at 24 weeks (risk ratio 3.69, 95% CI 2.37‐5.75; *P*<.001).

**Figure 5.**
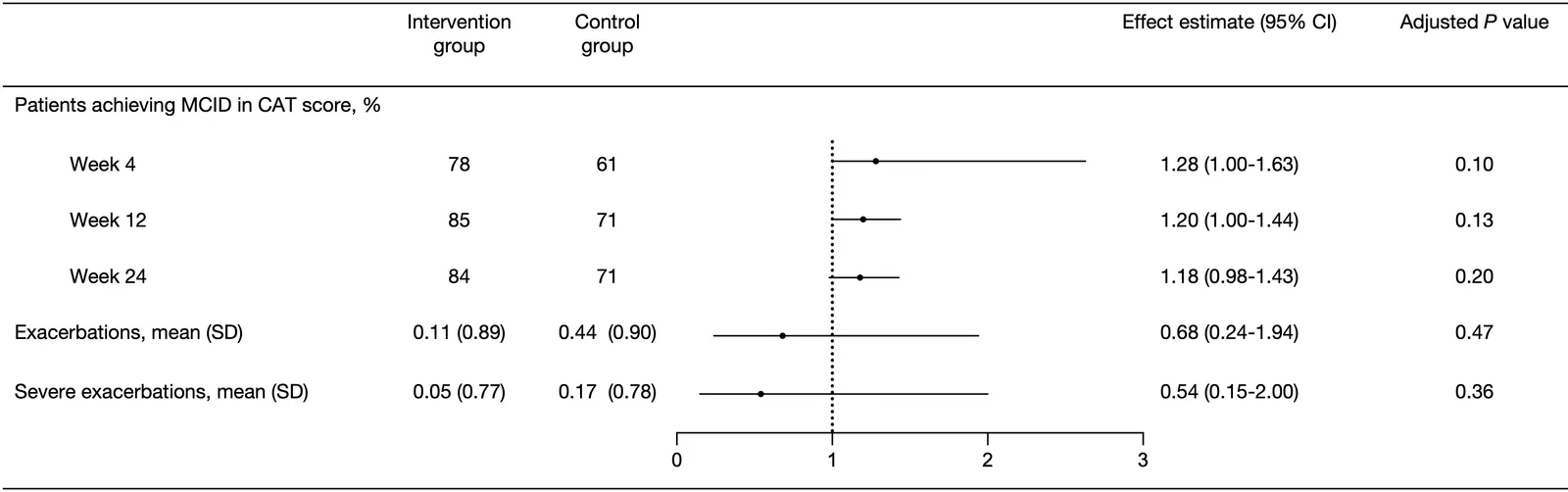
Effect of the intervention on COPD Assessment Test (CAT) and exacerbations. COPD: chronic obstructive pulmonary disease; MCID: minimal clinically important difference.

### Post Hoc Analyses (Exploratory Outcomes)

To further explore potential factors associated with adherence and clinical outcomes, we conducted post hoc exploratory correlation analyses, subgroup analyses, and multivariable regression.

Exploratory Pearson correlation analysis of the main outcome and baseline information showed that the average adherence of the intervention group was positively correlated with age (Pearson correlation coefficient, *r*=0.31; *P*=.03) and the presence of an acute exacerbation in the past year (*r*=0.225, *P*=.02), whereas no significant correlation was observed in the control group (*r*=0.049; *P*=.28).

Exploratory subgroup analyses for secondary outcomes were conducted according to age (<60 vs≥60 y), sex, GOLD (Global Initiative for Chronic Obstructive Lung Disease) stage, baseline TAI score (<46 vs ≥ 46), baseline mMRC grade (<2 vs≥2), history of exacerbations (0 vs ≥1), and electronic adherence (<80% vs ≥80%). For acute exacerbations at week 24, potential effect modification was observed by mMRC grade (*P*=.02), GOLD stage (*P*=.03), and adherence (*P*=.045). Patients with mMRC ≥2, GOLD stage 3, or adherence ≥80% showed greater reductions with the intervention (mean differences −0.68, 95% CI −1.10 to −0.26; *P*=.002; −1.75, 95% CI −2.72 to −0.78; *P*<.001; and −0.79, 95% CI −1.32 to −0.26; *P*=.004) compared with the respective comparator subgroups. No significant differential response pattern was observed for the other subgroups.

To identify characteristics associated with high adherence (adherence ≥80%), we performed univariate logistic regression analyses using baseline variables. Eleven variables were associated with high adherence (*P*<.20 or area under the curve [AUC]>0.6). Among these, GOLD group (AUC=0.631; *P*=.03) and BMI (AUC=0.614; *P*=.03) exhibited the highest predictive ability. A multivariable logistic model incorporating these 2 variables yielded a mean cross-validated AUC of 0.611 (SD 0.046) after 5-fold cross-validation, with a sensitivity of 31.7% and a specificity of 83.3%.

### Engagement and Satisfaction

The EIM demonstrated satisfactory performance in tracking medication adherence, but the completion rates of actively reported measures via the patient mobile app were notably low. During the 168-day follow-up period, the average completion rate for the daily CERT diary was only 7.0% (SD 23.7%), accompanied by a 2.3% (SD 16.5%) completion rate for body temperature logs and 5.3% (SD 23.2%) for oxygen saturation recordings. This finding may be attributed to the predominantly older adult study population, who generally exhibit lower proficiency and frequency in smartphone use. These results underscore the need for future digital therapeutic tools to incorporate more automated and user-friendly symptom monitoring modalities that minimize reliance on active patient input. Despite the low engagement with active app features, the end-of-study user satisfaction survey revealed favorable attitudes toward the digital therapeutic software. Among the 158 participants, 101 (63.9%) reported being “very satisfied” with the device, and 98 (62.0%) rated it as very convenient to use.

### Adverse Events

No adverse events were reported during the study.

## Discussion

This randomized controlled study provides evidence supporting the feasibility and preliminary effectiveness of a digital therapeutic intervention in Chinese patients with COPD. Participants receiving the digital therapeutic intervention integrating EIM with a digital management platform demonstrated significantly higher objectively measured inhaler adherence compared with electronic monitoring alone over the 24-week study period. However, improvements in clinical outcomes, including exacerbations, health-related quality of life, symptoms, and lung function, did not reach statistical significance. These findings suggest that digital therapeutics may be effective in improving medication-taking behavior, whereas larger and longer studies are needed to determine their impact on clinical outcomes.

Our results are consistent with previous studies in populations with asthma, in which electronic monitoring revealed adherence gaps not captured by patient self-report, emphasizing the value of objective, technology-enabled assessment in chronic airway disease management [21-24]. Despite the older age profile of patients with COPD relative to typical asthma cohorts, our results suggest that older adult patients can effectively engage with digital inhaler technologies. However, we observed a gradual decline in adherence over time despite digital support, suggesting that maintaining long-term behavioral change remains challenging. This finding highlights the value of regular, in-person clinical follow-up alongside digital tools to maintain long-term adherence [25]. Thus, we conducted exploratory telephone follow-ups in December 2025 to provide preliminary information regarding the longer-term implementation of the intervention. A total of 128 patients participated in this follow-up, of whom 91 continued to use inhaled medications, and 87 reported daily adherence in the past week. Future studies with predefined long-term follow-up protocols are needed to determine whether digital therapeutics can produce sustained behavioral and clinical benefits.

Notably, electronically measured adherence and self-reported adherence showed marked divergence. This discrepancy may be partly attributable to differences in the assessment windows: TAI assessed self-reported adherence during the preceding 1 week at prespecified follow-up visits, whereas EIM continuously captured inhaler use and summarized adherence over longer follow-up intervals. However, the magnitude of the observed difference suggests that the assessment window alone does not fully account for the divergence. Self-reported adherence is susceptible to recall bias and social desirability bias, as patients may overestimate their medication use during clinic visits. In addition, TAI includes items assessing medication-taking behavior as well as behavioral and perceptual factors related to nonadherence, whereas EIM primarily captures only the occurrence and timing of inhaler use. These findings highlight the complementary value of both objective monitoring and subjective self-report in assessing adherence and evaluating adherence-promoting strategies.

After adjusting for baseline values, the intervention group showed numerically better outcomes in exacerbation frequency, symptom scores, and lung function, though none reached statistical significance. These findings likely reflect the study being powered for adherence rather than clinical outcomes, moderate baseline disease severity, low exacerbation rates, and a relatively short 24-week follow-up. Additionally, CAT and mMRC scores improved over time in both groups. Given that both groups received regular follow-up and study participation itself may increase disease awareness and self-management behaviors, these improvements may partly reflect nonspecific effects, including increased attention or Hawthorne effects, rather than the isolated effect of the digital therapeutic intervention. Future studies incorporating attention-matched control conditions, factorial intervention designs, and longer follow-up are needed to better determine the independent contribution of specific digital therapeutic components. Although no statistically significant differences were observed in mental health measures, slightly higher PHQ-9 and GAD-7 scores in the intervention group suggest a need to monitor psychological burden associated with digital interventions. Previous literature indicates that higher eHealth literacy can improve quality of life, sleep quality, and resilience but may also be associated with increased anxiety and depression, highlighting the need for careful monitoring of potential adverse effects on mental health [26].

Subgroup analysis suggested that the effect of the digital therapeutic on acute exacerbations may be more pronounced in patients with greater symptom burden, more severe disease, and higher adherence. The observed reduction in acute exacerbations among patients with higher adherence suggests that improved medication adherence may represent one potential pathway through which digital therapeutics could influence clinical outcomes [27]. However, these findings should be interpreted cautiously, given the exploratory nature of the analyses, and require confirmation in future adequately powered studies. Multivariable regression analysis showed that the baseline GOLD grade and BMI modestly predicted high adherence. This indicates that, in clinical practice, adherence cannot be reliably predicted based solely on basic physiological or disease severity indicators. Dynamic factors during the intervention, such as patient engagement, social or family support, digital literacy, or behavioral patterns, may be stronger determinants of adherence than static baseline characteristics. Therefore, future research should integrate multidimensional assessments to better capture the complexity of medication adherence behavior and to elucidate the mechanisms underlying responses to digital therapeutics, including how specific functional designs influence patient behavioral change and how factors such as patients’ behavioral patterns, cognitive characteristics, and technology acceptance modulate intervention outcomes. By integrating behavioral science theories with digital technologies to develop more targeted intervention modules, we may further enhance the clinical efficacy of digital therapeutics and facilitate their evolution from a “one-size-fits-all” approach toward precision and personalized health management [28,29].

This study has several limitations. First, this was a single-center study recruiting through physician recommendation and patient self-referral. This may have introduced selection bias as individuals who agreed to participate were predominantly urban and educated and may have been more motivated and receptive to digital health technologies. In addition, the open-label design resulted in unequal levels of participant interaction between groups, and improvements in subjective outcomes may have been partly influenced by increased attention, behavioral reinforcement, or Hawthorne effects. These factors may limit the generalizability of the findings and may also have attenuated between-group differences in secondary clinical outcomes, as participants may have represented a more engaged subgroup of the population with COPD. Second, the EIM used is compatible only with pMDI devices and provides temporal reminders without guidance on inhalation technique. Third, the reliance on smartphone use among mostly older adult participants may exacerbate health inequities in underserved populations. Fourth, low completion rates of actively reported symptom diaries limited early detection of exacerbations. In addition, because the CERT diary was available only to the intervention group, the 2 groups differed with respect to the availability of systematic symptom data between scheduled visits. This differential access reflects the intended design of the digital therapeutic program; however, the very low completion rates in the intervention group suggest that the actual impact of this differential monitoring on clinical management may have been limited. Nonetheless, we cannot exclude the possibility that even sporadic symptom reports contributed to clinical awareness and earlier intervention in some cases. Fifth, the 6-month follow-up may be insufficient to capture long-term behavioral or clinical outcomes. Finally, health care resource use and cost-effectiveness data were not collected, precluding economic evaluation.

In summary, this study demonstrates that a digital therapeutic program can significantly improve inhaler adherence among Chinese patients with COPD, even in older adults, while the translation into short-term clinical benefit remains uncertain. These findings support the feasibility of digital therapeutics and highlight the need for longer-term studies incorporating automated, user-friendly monitoring and targeted behavioral strategies to maximize clinical impact. Future research should focus on identifying patient subgroups most likely to benefit and integrating behavioral science with digital health design to optimize personalized COPD management [7,28,29].

## Supplementary material

10.2196/98870 Checklist 1 CONSORT checklist.

## Abbreviations

AUC: area under the curve
CAT: COPD Assessment Test
CERT: COPD Exacerbation Recognition Tool
CONSORT: Consolidated Standards of Reporting Trials
COPD: chronic obstructive pulmonary disease
DIGI-COPD: Effectiveness of a Multi-Component Digital Therapeutic Program on Inhaled Medication Adherence in COPD
EIM: electronic inhaler monitoring
FEV_1_/FVC: forced expiratory volume in 1 second/forced vital capacity
GAD-7: Generalized Anxiety Disorder-7
GOLD: Global Initiative for Chronic Obstructive Lung Disease
mMRC: modified Medical Research Council
PHQ-9: Patient Health Questionnaire-9
pMDI: pressurized metered-dose inhaler
SGRQ-C: St George’s Respiratory Questionnaire for Chronic Obstructive Pulmonary Disease
TAI: Test of Adherence to Inhalers
